# A Phase 1 Double-Blinded Trial to Evaluate Safety, Immunogenicity, and Dosing of Measles-Vectored Chikungunya Virus Vaccine (MV-CHIK) in Healthy Adults

**DOI:** 10.1093/infdis/jiaf571

**Published:** 2025-11-28

**Authors:** Patricia Winokur, Theresa E Hegmann, Hana M El Sahly, Evan J Anderson, DMID 15-0038 Study Group, Nancy Wagner, Tom Kaufman, Wendy A Keitel, Robert L Atmar, Jumi Yi, Inci Yildirim, Carol Kao, Etza Peters, Kathy Stephens, Christina Rostad, Walla Dempsey, Venus Shahamatdar, Tatiana Beresney, Kay Tomashek

**Affiliations:** University of Iowa, Department of Internal Medicine, Iowa City, Iowa, USA; University of Iowa, Department of Physician Assistant Studies, Iowa City, Iowa, USA; Baylor College of Medicine, Departments of Molecular Virology and Microbiology and Medicine, Baylor College of Medicine, Houston, Texas, USA; Emory University, Department of Medicine and Department of Pediatrics, Atlanta, Georgia

**Keywords:** chikungunya, vaccine, phase 1 trial, measles-vectored vaccine

## Abstract

This study of a measles-vectored vaccine for chikungunya virus (MV-CHIK) was a Phase 1 randomized, double-blinded, placebo-controlled trial with varying intervals between the 2 doses. The 6 cohorts each had 30 subjects, of which 25 received MV-CHIK and 5 received placebo. The 5 × 10^5^ TCID50 dose was superior to the 5 × 10^4^ TCID50 dose, and longer dosing intervals resulted in higher titers in the high dose groups. Mild to moderate injection site and systemic reactogenicity was common but brief. There was no evidence of prolonged vaccine-related arthralgia.

## Teaser

This Phase 1 clinical trial evaluated a measles-vectored chikungunya vaccine. The vaccine elicited good antibody titers and was well tolerated. Two doses of the higher titered vaccine and longer intervals between doses elicited the highest antibody titers.

Chikungunya virus (CHIKV) is a mosquito-borne pathogen that causes an acute febrile illness in humans, frequently accompanied by severe, debilitating arthralgia. Although mortality is rare, over 50% of those infected suffer from prolonged arthralgias that can last for months to years [1, 2]. Historically, CHIKV infections have been restricted to a relatively limited number of tropical areas. However, adaptation of CHIKV to allow transmission by the *Aedes albopictus* mosquito, in addition to continued efficient transmission by *Aedes aegypti*, led to expansion of its geographic distribution, with endemic and epidemic disease now occurring throughout the Americas, Europe, and much of Asia and Africa [1, 3–5].

The vaccine evaluated in this study (MV-CHIK) is a live-attenuated recombinant, measles-vectored vaccine based on the Schwartz vaccine strain of measles virus. The vector has been modified to carry CHIKV structural genes to produce a replication-competent virus that expresses both measles and chikungunya proteins [6–9]. The current study expands upon the results of a 2019 European Phase 2 randomized, double-blinded, placebo-, and active-controlled study of the MV-CHIK vaccine by providing a more extensive evaluation of the impact of dosing interval on immune response and durability [9].

## METHODS

### Study Design and Safety Procedures

This Phase 1 randomized, double-blinded, placebo-controlled trial was designed to evaluate safety, tolerability, immunogenicity and optimal dose and dosing interval for the MV-CHIK vaccine candidate. It was conducted at three centers in the United States from June 2017 to January 2019. Planned enrollment was 180 healthy adult subjects aged 18 to 45 years, inclusive, randomized into 6 cohorts to evaluate a low dose versus high dose (5 × 10^4^ or 5 × 10^5^ of tissue culture infective dose-50% [TCID50]), as well as 3 dosing intervals (second dose administered on Days 29, 85, or 169). Screening included evaluation of medical history, travel history to countries with known CHIKV circulation, medications, physical examination, and safety laboratory evaluations. Detailed inclusion and exclusion criteria are provided at www.ClinicalTrials.gov (NCT03028441).

The study was sponsored by the National Institute of Allergy and Infectious Diseases and approved by each local institutional review board. All participants gave written informed consent prior to any study procedures. A total of 307 subjects were screened and 180 were enrolled, with 30 randomized to each of cohorts 1–6, using a 5 active to 1 placebo ratio (Supplementary Figure 1, CONSORT Flow Diagram). Blood and urine safety laboratory analyses were performed prior to and on Day 15 following each study injection. Solicited local and systemic adverse events (AEs) were collected through Day 15 following each injection, unsolicited nonserious AEs through Day 29 following each injection, and serious adverse events (SAEs) and AEs of special interest (AESIs) through 6 months following the last injection.

### Study Vaccine and Control Products

MV-CHIK vaccine was supplied by Themis Bioscience GmbH. Sterile water for injection (WFI, USP) was used as vaccine diluent and normal saline was used as the placebo injection; both were supplied by Fisher BioServices. MV-CHIK was formulated as a lyophilized vaccine product containing hydrolyzed gelatin from porcine origin as an excipient. Each single use glass 2 mL vial contained 5 × 10^4^ (+/−0.5 log) TCID50/dose or 5 × 10^5^ (+/−0.5 log) TCID50/dose. The vaccine was stored frozen at −20°C (+/−5°C) until reconstitution by the site research pharmacist by adding 0.4 mL WFI.

### Planned Outcomes

The primary objectives were to evaluate the safety and tolerability of MV-CHIK vaccine following two intramuscular injections, and to assess the CHIKV serum plaque reduction neutralization test (PRNT50) antibody responses to low and high dose MV-CHIK or placebo on Day 29 following the first dose. Antibody responses also were analyzed by ELISA methods. Full details regarding all primary and secondary outcome measures are provided at www.ClinicalTrials.gov (NCT03028441).

### Statistical Analysis

All continuous variables were summarized using the following descriptive statistics: n (nonmissing sample size), mean, standard deviation, median, maximum, and minimum. The frequency and percentages (based on the nonmissing sample size) of observed levels were reported for all categorical measures. No adjustment for multiple comparisons was performed in this Phase 1 study.

Baseline and demographic characteristics were summarized overall and by cohort.

Immunogenicity data summaries and analyses for primary and secondary endpoints were presented for the immunogenicity and intention-to-treat populations. Immunogenicity values that were below the lower limit of quantification (LLOQ) were replaced by half the LLOQ for analysis. For PRNT50 assays, the LLOQ was 10 and for ELISA assays, the LLOQ was 14.70. Anti-CHIKV PRNT50 and ELISA antibody titers were summarized by dose group (low dose, high dose, and placebo) for all subjects pooled across vaccination schedules, and by dose within the dose schedule subgroups. Summaries were presented for each time point relative to vaccination (prevaccination dose 1, 29 days post vaccination dose 1, prevaccination 2, and 15, 29, 85, 169 days postvaccination dose 2) and at the peak titer (from any postvaccination time point).

Individuals with missing baseline titers were not included in summaries of geometric mean fold rise (GMFR) or ≥4-fold increase seroconversion rates. Clopper–Pearson CIs were calculated for percentages. Per the plan for statistical analysis, differences with 95% CIs for geometric mean titer (GMT) and GMFR by dose level were calculated using a *t*-test on log_10_−transformed values and were exponentiated for presentation as ratios between high dose MV-CHIK and low dose MV-CHIK. Exact unconditional 95% CIs for differences in proportions of seroconverting or seropositive subjects were calculated by the Score method [10]. *P*-values for comparisons of GMT and GMFR by dose schedule were computed using ANOVA; *P*-values for seroconversion and seropositivity rates were computed using Fisher's exact test.

## RESULTS

### Study Population

Participants were enrolled between June 2017 and March 2018. Twenty-one subjects left the study before completion: 9 (12%) from low dose groups, 10 (13%) from high dose groups, and 2 (7%) from the placebo group (Supplementary Figure 1). Eighty-seven percent (156 of 180) subjects received both doses of vaccine. One hundred fifty-nine subjects (88%) completed the study through follow-up, and 176 (98%) completed the blood draw at Day 29 after the first vaccination for the primary analysis. Overall, the majority of subjects were female (58%), not Hispanic or Latino (91%), and White (68%). Ethnicity, race, and the percentage of females were similar across groups. The median age for the subjects was 28 years (range: 18 to 45 years) with a mean age of 29.5.

### Safety and Reactogenicity

Solicited AEs were common but mainly mild to moderate in both dose groups, with 83% (125 of 150) of individuals who received any dose of MV-CHIK experiencing a solicited AE through 15 days post either dose (Supplementary Table 1). There were no major differences identified between the first and second doses for solicited AEs. The most common solicited systemic AEs included headache, fatigue, and malaise. There was no statistically significant difference between the low and high dose groups for solicited systemic AEs (*P* = .504). For injection site AEs, there was a statistically significant difference between dosing groups: 85% of the high dose group reported experiencing an injection site AE, versus only 59% of the low dose group (*P* < .001). No serious injection site events were reported in either group. Supplementary Figures 2 and 3 summarize the maximum severity of solicited systemic and injection site AEs after either vaccination, respectively.

Unsolicited AEs were reported in 47% of subjects in the low dose group and 52% of subjects in the high dose group. There were five SAEs in four subjects; none were considered related to study product. Two individuals in the placebo group experienced an AESI (one with arthralgia that was not considered to be related to study product and one with intermittent joint pain that was considered related). No subjects in the vaccine groups reported an AESI.

### Immunogenicity

**Figure 1.**
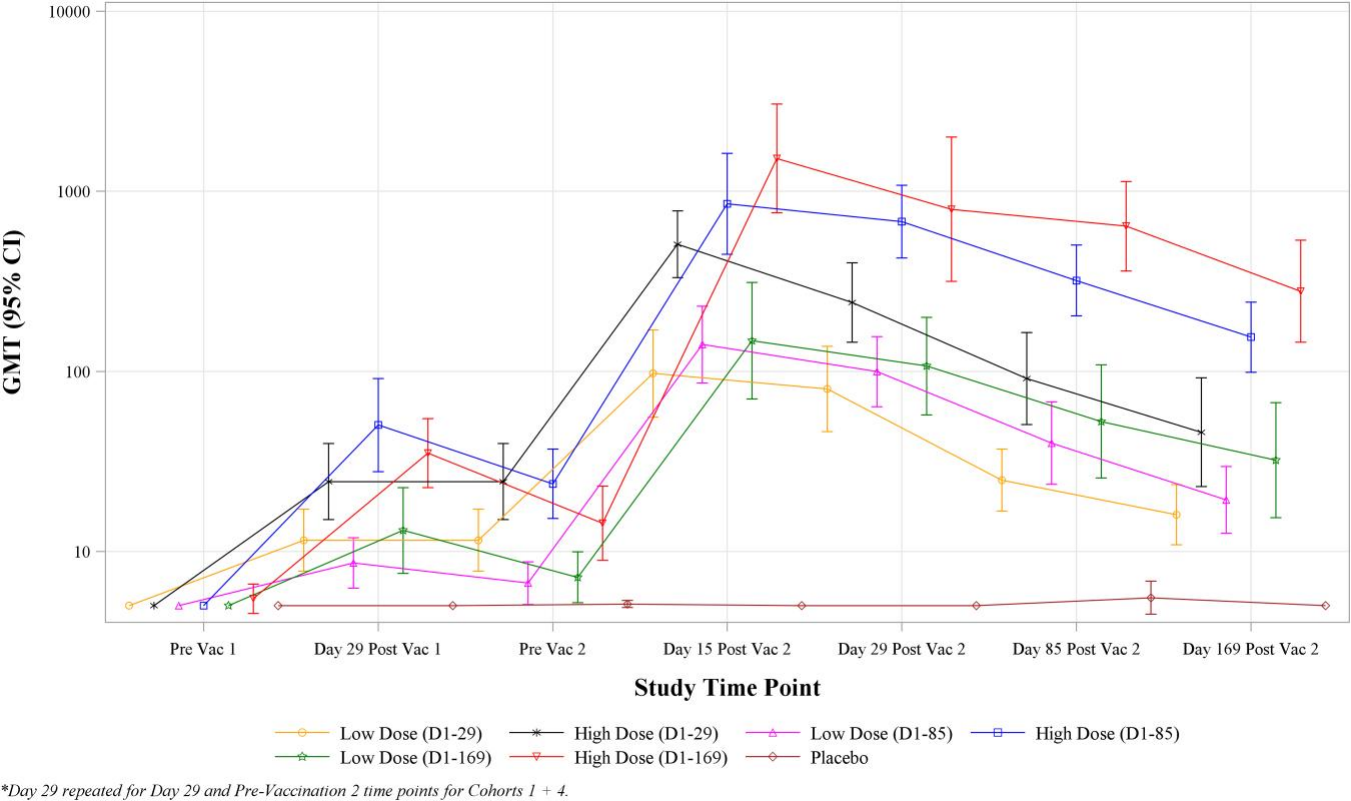
Time trend plots of geometric mean anti-CHIKV PRNT50 titer by time point and study group—immunogenicity population.

Figure 1 and Supplementary Table 2 show antibody kinetics data for geometric mean anti-CHIKV PRNT50 titer for each cohort across each study time point. At Day 29 (the primary immunogenicity endpoint), the GMT was 10.9 (95% CI: 8.6, 13.9) for low dose, 35.2 (95% CI: 26.3, 47.1) for high dose and 5.0 for Placebo with a ratio between high dose and low dose GMTs of 3.2 (95% CI: 2.2, 4.7), *P* < .001. ELISA results followed very similar patterns and are provided in Supplementary Table 3.

**Figure 2.**
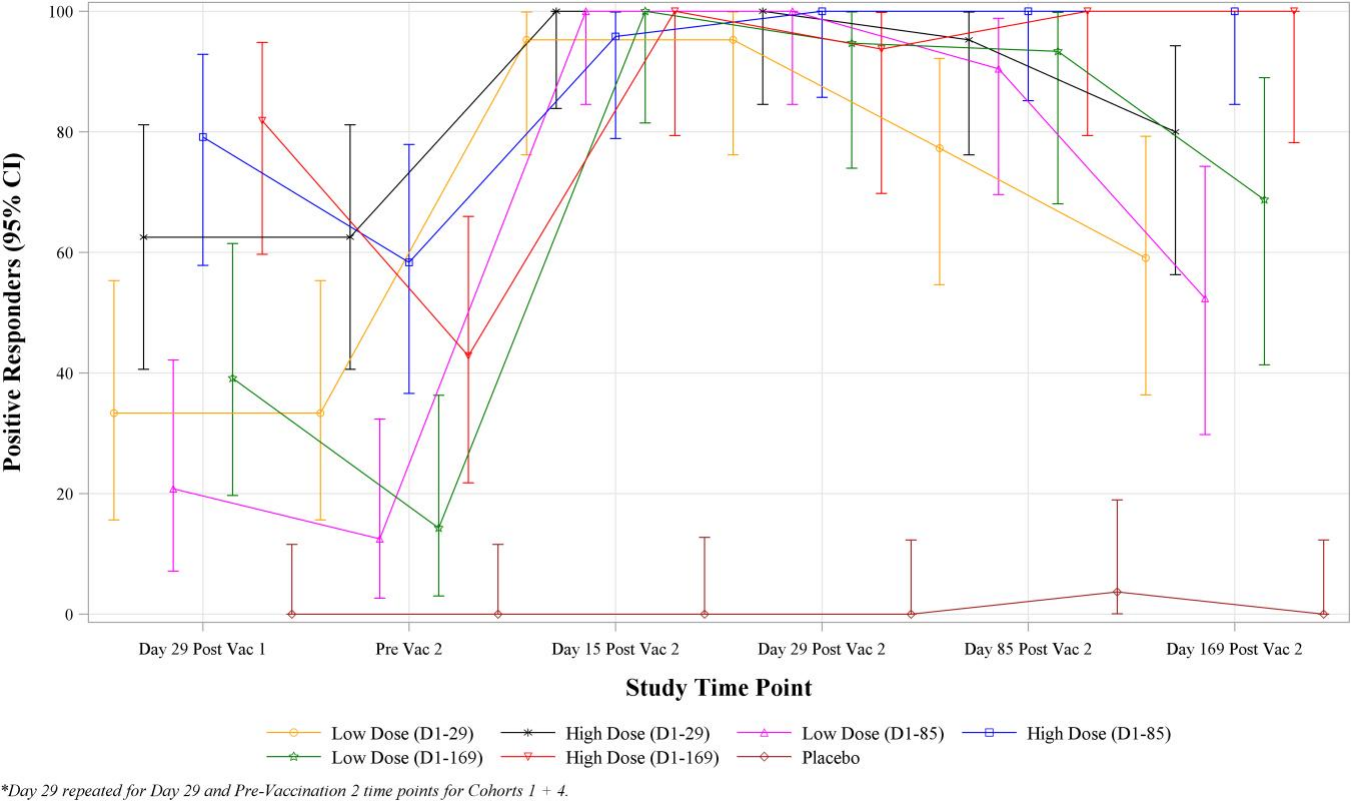
Time trend plots of percent anti-CHIKV PRNT50 seroconversion by time point and study group—immunogenicity population.

On average, seroconversion at Day 29 occurred in 31% of individuals (95% CI: 21%, 43%) in the low dose groups and 74% of those in the high dose groups (95% CI: 62%, 84%), *P* < .001. Thus, a single high dose of MV-CHIK achieved a superior immune response compared to a single low dose. Figure 2 provides a time trend plot showing seroconversion rates by time point and study group in the immunogenicity population. Again, the ELISA results are similar, and no subject in the placebo group met criteria for seropositivity or seroconversion (Supplementary Table 3).

### Effect of Immunization Intervals

A secondary objective for this study was to compare the 3 dosing intervals by assessing the Day 29 postsecond vaccination PRNT responses in subjects who received immunizations on Days 1 and 29, Days 1 and 85, or Days 1 and 169 in either the low dose or high dose groups (Supplementary Table 4). For the low dose groups, the 3 different dosing intervals were not associated with statistically significant differences in PRNT50 GMTs or seroconversion rates. In the high dose groups, the PRNT50 GMTs were found to be significantly different between the 3 dosing schedules (*P* = .009); specifically 241.0 (95% CI: 145.0, 400.4) for subjects vaccinated on Days 1 and 29, 678.1 (95% CI: 426.3, 1078.5) for subjects vaccinated on Days 1 and 85, and 794.8 (95% CI: 316.2, 1998.0) for subjects vaccinated on Days 1 and 169. However, seroconversion rates across the three dosing intervals in the high dose groups showed no statistically significant difference, with 94–100% of subjects seroconverting by Day 29 following the second dose.

## DISCUSSION

In summary, the 5 × 10^5^ TCID50 dose appeared to be superior to the lower dose of 5 × 10^4^ TCID50 and higher titers were achieved after a second dose, and with a longer interval between the two immunizations in the high dose group. Previous trials of vaccines for various pathogens have demonstrated a similar potential for obtaining an improved immune response by delaying the timing of the boost [11, 12]. High rates of seroconversion were seen with both dosages at 29 days after the second dose (94–100%). However, the durability of the immune response appeared to be superior in the high dose groups (data not shown).

Similar to the Phase 2 European study, this study showed the MV-CHIK vaccine to be safe and well tolerated [9]. Both studies showed that solicited AEs were common; in the current study over 80% of individuals had at least one solicited AE. However, the majority was mild to moderate in severity and resolved within 1–3 days. This study did show that the high dose was associated with more injection site tenderness and pain, but again the severity was mild to moderate in nature.

Joint symptoms that lasted longer than 96 hours were tracked in this study as AESIs. No vaccine recipient experienced prolonged arthralgia. Given the significant AE rates of prolonged joint pain associated with one of the currently licensed Chikungunya vaccines (Ixchiq®) [13, 14], the absence of AESIs in subjects who received MV-CHIK vaccine in this study is promising.

In summary, this study showed a stronger immune response after 2 doses of the high dose formulation of MV-CHIK vaccine compared with the lower dose. Notably, this study also demonstrated that longer dosing intervals were associated with progressively higher titers in the high dose groups. Though longer dose intervals are often advantageous, in an outbreak setting shorter dose intervals may allow more rapid acquisition of individual and community protection. As the MV vaccine platform use expands, opportunities exist to evaluate the influence of pre-existing measles virus titers on MV-platform vaccine performance as well as the impact of platform vaccine on measles virus immune responses.

## Supplementary Material

jiaf571_Supplementary_Data

## Supplementary Data

Supplementary materials are available at *The Journal of Infectious Diseases* online (http://jid.oxfordjournals.org/). Supplementary materials consist of data provided by the author that are published to benefit the reader. The posted materials are not copyedited. The contents of all supplementary data are the sole responsibility of the authors. Questions or messages regarding errors should be addressed to the author.
