# Supplementary material for: A Phase 1 Double-Blinded Trial to Evaluate Safety, Immunogenicity, and Dosing of Measles-Vectored Chikungunya Virus Vaccine (MV-CHIK) in Healthy Adults
Source: J Infect Dis. 2025 Nov 28;233(3):e641–5. doi: 10.1093/infdis/jiaf571 (PMC13017142; doi:10.1093/infdis/jiaf571)
Supplement: jiaf571_Supplementary_Data [file jiaf571_supplementary_data.zip › Supplementary Figure and Table Legends.docx]

**Supplementary Figure and Table Legends**

**Supplementary Figure 1**: CONSORT Flow Diagram

**Supplementary Figure 2**: Maximum Severity of Solicited Systemic Symptoms per Subject by Day Post-Vaccination and Dose, Post Either Vaccination (Safety Population)

**Supplementary Figure 3**: Maximum Severity of Solicited Local Symptoms per Subject by Day Post-Vaccination and Dose, Post Either Vaccination (Safety Population)

**Supplementary Table 1:** Number and Percentage of Subjects Experiencing Solicited Events with 95% Confidence Intervals by Symptom and Dose Through 15 Days Post Either Vaccination (Safety Population)

**Supplementary Table 2:** Summaries of Anti-CHIKV PRNT50 Antibody by Dose (Immunogenicity Population)

**Supplementary Table 3:** Summaries of Anti-CHIKV ELISA Antibody by Dose (Immunogenicity Population)

**Supplementary Table 4:** Anti-CHIKV PRNT50 Antibody Results on Day 29 After Second Vaccination by Dose and Schedule (Immunogenicity Population)
