# Supplementary material for: A Phase 1 Double-Blinded Trial to Evaluate Safety, Immunogenicity, and Dosing of Measles-Vectored Chikungunya Virus Vaccine (MV-CHIK) in Healthy Adults
Source: J Infect Dis. 2025 Nov 28;233(3):e641–5. doi: 10.1093/infdis/jiaf571 (PMC13017142; doi:10.1093/infdis/jiaf571)
Supplement: jiaf571_Supplementary_Data [file jiaf571_supplementary_data.zip › Supplementary Table 1.docx]

Supplementary Table 1: Number and Percentage of Subjects Experiencing Solicited Events with 95% Confidence Intervals by Symptom and Dose Through 15 Days Post Either Vaccination (Safety Population)

| **Either Vaccination** | | | | | | | | | | | | | |  |
| --- | --- | --- | --- | --- | --- | --- | --- | --- | --- | --- | --- | --- | --- | --- |
|  | **Low Dose MV-CHIK (N=75)** | | | **High Dose MV-CHIK (N=75)** | | | **All MV-CHIK (N=150)** | | | **All Placebo (N=30)** | | |  |  |
|  | **n** | **%** | **95% CI** | **n** | **%** | **95% CI** | **n** | **%** | **95% CI** | **n** | **%** | **95% CI** | **P-value^a^ Low dose *vs* High dose** |  |
| Any Symptom | 58 | 77 | (66, 86) | 67 | 89 | (80, 95) | 125 | 83 | (76, 89) | 22 | 73 | (54, 88) | 0.078 |  |
| Any Systemic Symptom | 48 | 64 | (52, 75) | 43 | 57 | (45, 69) | 91 | 61 | (52, 69) | 19 | 63 | (44, 80) | 0.504 |  |
| Fever | 4 | 5 | (1, 13) | 3 | 4 | (<1, 11) | 7 | 5 | (2, 9) | 2 | 7 | (<1, 22) | 1.000 |  |
| Headache | 39 | 52 | (40, 64) | 27 | 36 | (25, 48) | 66 | 44 | (36, 52) | 15 | 50 | (31, 69) | 0.070 |  |
| Fatigue | 29 | 39 | (28, 51) | 29 | 39 | (28, 51) | 58 | 39 | (31, 47) | 8 | 27 | (12, 46) | 1.000 |  |
| Malaise | 23 | 31 | (21, 42) | 13 | 17 | (10, 28) | 36 | 24 | (17, 32) | 7 | 23 | (10, 42) | 0.084 |  |
| Myalgia | 15 | 20 | (12, 31) | 12 | 16 | (9, 26) | 27 | 18 | (12, 25) | 8 | 27 | (12, 46) | 0.671 |  |
| Joint Pain | 8 | 11 | (5, 20) | 8 | 11 | (5, 20) | 16 | 11 | (6, 17) | 3 | 10 | (2, 27) | 1.000 |  |
| Chills | 8 | 11 | (5, 20) | 4 | 5 | (1, 13) | 12 | 8 | (4, 14) | 4 | 13 | (4, 31) | 0.367 |  |
| Underarm pain | 4 | 5 | (1, 13) | 2 | 3 | (<1, 9) | 6 | 4 | (1, 9) | 0 | 0 | (0, 12) | 0.681 |  |
| Underarm swelling | 0 | 0 | (0, 5) | 1 | 1 | (<1, 7) | 1 | <1 | (<1, 4) | 0 | 0 | (0, 12) | 1.000 |  |
| Anorexia | 5 | 7 | (2, 15) | 8 | 11 | (5, 20) | 13 | 9 | (5, 14) | 3 | 10 | (2, 27) | 0.563 |  |
| Nausea | 13 | 17 | (10, 28) | 13 | 17 | (10, 28) | 26 | 17 | (12, 24) | 4 | 13 | (4, 31) | 1.000 |  |
| Vomiting | 3 | 4 | (<1, 11) | 1 | 1 | (<1, 7) | 4 | 3 | (<1, 7) | 1 | 3 | (<1, 17) | 0.620 |  |
| Any Local Symptom | 44 | 59 | (47, 70) | 64 | 85 | (75, 92) | 108 | 72 | (64, 79) | 15 | 50 | (31, 69) | <0.001 |  |
| Pain – experienced without touching the injection site | 19 | 25 | (16, 37) | 37 | 49 | (38, 61) | 56 | 37 | (30, 46) | 4 | 13 | (4, 31) | 0.004 |  |
| Tenderness | 34 | 45 | (34, 57) | 56 | 75 | (63, 84) | 90 | 60 | (52, 68) | 10 | 33 | (17, 53) | <0.001 |  |
| Erythema/Redness (Measured) | 0 | 0 | (0, 5) | 2 | 3 | (<1, 9) | 2 | 1 | (<1, 5) | 0 | 0 | (0, 12) | 0.497 |  |
| Erythema/Redness (Functional) | | 20 | 27 | (17, 38) | 16 | 21 | (13, 32) | 36 | 24 | (17, 32) | 10 | 33 | (17, 53) | 0.567 |
| Induration/Swelling (Measured) | | 0 | 0 | (0, 5) | 0 | 0 | (0, 5) | 0 | 0 | (0, 2) | 0 | 0 | (0, 12) | - |
| Induration/Swelling (Functional) | | 4 | 5 | (1, 13) | 3 | 4 | (<1, 11) | 7 | 5 | (2, 9) | 6 | 20 | (8, 39) | 1.000 |
| Ecchymosis/Bruising (Measured) | | 0 | 0 | (0, 5) | 0 | 0 | (0, 5) | 0 | 0 | (0, 2) | 0 | 0 | (0, 12) | - |
| Warmth at site of injection | | 6 | 8 | (3, 17) | 4 | 5 | (1, 13) | 10 | 7 | (3, 12) | 2 | 7 | (<1, 22) | 0.745 |
| Pruritus | | 2 | 3 | (<1, 9) | 3 | 4 | (<1, 11) | 5 | 3 | (1, 8) | 1 | 3 | (<1, 17) | 1.000 |

Notes: N=number of subjects in the Safety Analysis Population who received either Vaccination. Denominators for percentages are the number of subjects with non-missing data for the specified symptom. Exact 95% CIs for proportions are calculated using the Clopper-Pearson method.

ap-value for comparison of low dose *vs* high dose using Fisher’s exact test.
