# Supplementary material for: A Phase 1 Double-Blinded Trial to Evaluate Safety, Immunogenicity, and Dosing of Measles-Vectored Chikungunya Virus Vaccine (MV-CHIK) in Healthy Adults
Source: J Infect Dis. 2025 Nov 28;233(3):e641–5. doi: 10.1093/infdis/jiaf571 (PMC13017142; doi:10.1093/infdis/jiaf571)
Supplement: jiaf571_Supplementary_Data [file jiaf571_supplementary_data.zip › Supplementary Table 2.docx]

Supplementary Table 2: Summaries of Anti-CHIKV PRNT50 Antibody by Dose (Immunogenicity Population)

| **Time Point** | **Statistic** | **Low Dose MV-CHIK (N=73)** | **High Dose MV-CHIK (N=71)** | **All Placebo (N=30)** | **Difference^†^** |
| --- | --- | --- | --- | --- | --- |
| Day 1 (Pre-vaccine) | n | 73 | 71 | 30 | - |
|  | GMT (95% CI) | 5.0 (-) | 5.1 (4.9, 5.5) | 5.0 (-) | 1.0 (1.0, 1.1) |
|  | Seropositive % (95% CI), >= 10 titer | 0 (0, 5) | 1 (<1, 8) | 0 (0, 12) | 1 (-4, 8) |
| Day 29 Post vaccination 1 | n | 71 | 70 | 30 | - |
|  | GMT (95% CI) | 10.9 (8.6, 13.9) | 35.2 (26.3, 47.1) | 5.0 (-) | 3.2 (2.2, 4.7) |
|  | GMFR (95% CI) | 2.2 (1.7, 2.8) | 6.8 (5.0, 9.3) | 1.0 (-) | 3.1 (2.1, 4.6) |
|  | Seropositive % (95% CI), >= 10 titer | 52 (40, 64) | 87 (77, 94) | 0 (0, 12) | 35 (19, 49) |
|  | Seroconversion % (95% CI), >= 4-fold rise | 31 (21, 43) | 74 (62, 84) | 0 (0, 12) | 43 (27, 58) |
| Pre-vaccination Dose 2^*^ | n | 69 | 69 | 30 | - |
|  | GMT (95% CI) | 8.3 (6.8, 10.0) | 20.6 (15.9, 26.8) | 5.1 (4.9, 5.4) | 2.5 (1.8, 3.4) |
|  | GMFR (95% CI) | 1.7 (1.4, 2.0) | 4.0 (3.0, 5.3) | 1.0 (1.0, 1.1) | 2.4 (1.7, 3.4) |
|  | Seropositive % (95% CI), >= 10 titer | 36 (25, 49) | 81 (70, 90) | 3 (<1, 17) | 45 (29, 59) |
|  | Seroconversion % (95% CI), >= 4-fold rise | 20 (12, 32) | 55 (43, 67) | 0 (0, 12) | 35 (17, 49) |
| Day 15 Post vaccination 2 | n | 61 | 61 | 27 | - |
|  | GMT (95% CI) | 126.0 (91.2, 174.2) | 831.2 (589.7, 1171.5) | 5.0 (-) | 6.6 (4.1, 10.5) |
|  | GMFR (95% CI) | 25.2 (18.2, 34.8) | 166.2 (117.9, 234.3) | 1.0 (-) | 6.6 (4.1, 10.5) |
|  | Seropositive % (95% CI), >= 10 titer | 98 (91, >99) | 98 (91, >99) | 0 (0, 13) | 0 (-7, 7) |
|  | Seroconversion % (95% CI), >= 4-fold rise | 98 (91, >99) | 98 (91, >99) | 0 (0, 13) | 0 (-7, 7) |
| Day 29 Post vaccination 2 | n | 62 | 62 | 28 | - |
|  | GMT (95% CI) | 94.6 (70.6, 126.9) | 489.4 (344.0, 696.3) | 5.0 (-) | 5.2 (3.3, 8.1) |
|  | GMFR (95% CI) | 18.9 (14.1, 25.4) | 97.9 (68.8, 139.3) | 1.0 (-) | 5.2 (3.3, 8.1) |
|  | Seropositive % (95% CI), >= 10 titer | 100 (94, 100) | 98 (91, >99) | 0 (0, 12) | -2 (-9, 4) |
|  | Seroconversion % (95% CI), >= 4-fold rise | 97 (89, >99) | 98 (91, >99) | 0 (0, 12) | 2 (-6, 10) |
| Day 85 Post vaccination 2 | n | 58 | 60 | 27 | - |
|  | GMT (95% CI) | 35.9 (26.7, 48.4) | 248.2 (174.0, 354.0) | 5.5 (4.5, 6.8) | 6.9 (4.4, 10.9) |
|  | GMFR (95% CI) | 7.2 (5.3, 9.7) | 49.6 (34.8, 70.8) | 1.1 (0.9, 1.4) | 6.9 (4.4, 10.9) |
|  | Seropositive % (95% CI), >= 10 titer | 97 (88, >99) | 98 (91, >99) | 4 (<1, 19) | 2 (-6, 10) |
|  | Seroconversion % (95% CI), >= 4-fold rise | 86 (75, 94) | 98 (91, >99) | 4 (<1, 19) | 12 (3, 24) |
| Day 169 Post vaccination 2 | n | 59 | 57 | 28 | - |
|  | GMT (95% CI) | 20.7 (15.7, 27.4) | 118.1 (80.9, 172.2) | 5.0 (-) | 5.7 (3.6, 9.0) |
|  | GMFR (95% CI) | 4.1 (3.1, 5.5) | 23.6 (16.2, 34.4) | 1.0 (-) | 5.7 (3.6, 9.0) |
|  | Seropositive % (95% CI), >= 10 titer | 85 (73, 93) | 98 (91, >99) | 0 (0, 12) | 13 (3, 26) |
|  | Seroconversion % (95% CI), >= 4-fold rise | 59 (46, 72) | 93 (83, 98) | 0 (0, 12) | 34 (18, 48) |
| Peak Titer | n | 72 | 71 | 30 | - |
|  | GMT (95% CI) | 102.8 (73.2, 144.3) | 609.5 (418.3, 888.2) | 5.6 (4.6, 6.8) | 5.9 (3.6, 9.8) |
|  | GMFR (95% CI) | 20.6 (14.6, 28.9) | 118.4 (79.5, 176.4) | 1.1 (0.9, 1.4) | 5.8 (3.4, 9.7) |
|  | Seropositive % (95% CI), >= 10 titer | 94 (86, 98) | 100 (95, 100) | 7 (<1, 22) | 6 (<1, 14) |
|  | Seroconversion % (95% CI), >= 4-fold rise | 92 (83, 97) | 97 (90, >99) | 3 (<1, 17) | 6 (-3, 15) |

Notes: N=Number of subjects in the Immunogenicity Population. n=Number of subjects with non-missing endpoint data.

*Day of Second Dose MV-CHIK vaccine, Sample taken pre-dose. For Cohorts 1 and 4, Day 29 Post vaccination 1 and Pre-vaccination Dose 2 are the same study time point and the data appear in both rows.

†Difference = difference in the GMT, GMFR, Seropositive and Seroconversion rates, with 95% CI of the difference between Low Dose and High Dose MV-CHIK.

Exact 95% CIs for proportions are calculated using the Clopper-Pearson method. For the difference in proportions, exact unconditional confidence limits are calculated based on score statistics. 95% CIs are based on the t-test for differences in GMT and GMFR results.
