# Supplementary material for: A Phase 1 Double-Blinded Trial to Evaluate Safety, Immunogenicity, and Dosing of Measles-Vectored Chikungunya Virus Vaccine (MV-CHIK) in Healthy Adults
Source: J Infect Dis. 2025 Nov 28;233(3):e641–5. doi: 10.1093/infdis/jiaf571 (PMC13017142; doi:10.1093/infdis/jiaf571)
Supplement: jiaf571_Supplementary_Data [file jiaf571_supplementary_data.zip › Supplementary Table 3.docx]

Supplementary Table 3: Summaries of Anti-CHIKV ELISA Antibody by Dose (Immunogenicity Population)

| **Time Point** | **Statistic** | **Low Dose MV-CHIK (N=75)** | **High Dose MV-CHIK (N=75)** | **All Placebo (N=30)** | **Difference^†^** |
| --- | --- | --- | --- | --- | --- |
| Day 1 (Pre-vaccine) | n | 73 | 71 | 30 | - |
|  | GMT (95% CI) | 7.5 (7.2, 7.7) | 7.4 (-) | 7.4 (-) | 1.0 (0.9, 1.0) |
|  | Seropositive % (95% CI), >= 10 titer | 1 (<1, 7) | 0 (0, 5) | 0 (0, 12) | -1 (-7, 4) |
| Day 29 Post vaccination 1 | n | 70 | 70 | 30 | - |
|  | GMT (95% CI) | 10.2 (8.6, 12.2) | 44.4 (34.3, 57.5) | 7.4 (-) | 4.3 (3.2, 5.9) |
|  | GMFR (95% CI) | 1.4 (1.1, 1.6) | 6.0 (4.7, 7.8) | 1.0 (-) | 4.4 (3.3, 6.0) |
|  | Seropositive % (95% CI), >= 10 titer | 21 (13, 33) | 86 (75, 93) | 0 (0, 12) | 64 (50, 76) |
|  | Seroconversion % (95% CI), >= 4-fold rise | 10 (4, 20) | 67 (55, 78) | 0 (0, 12) | 57 (42, 69) |
| Pre-vaccination Dose 2^*^ | n | 68 | 69 | 30 | - |
|  | GMT (95% CI) | 9.8 (8.4, 11.3) | 35.2 (26.7, 46.5) | 7.4 (-) | 3.6 (2.6, 4.9) |
|  | GMFR (95% CI) | 1.3 (1.1, 1.5) | 4.8 (3.6, 6.3) | 1.0 (-) | 3.7 (2.7, 5.0) |
|  | Seropositive % (95% CI), >= 10 titer | 21 (12, 32) | 78 (67, 87) | 0 (0, 12) | 58 (42, 71) |
|  | Seroconversion % (95% CI), >= 4-fold rise | 7 (2, 16) | 61 (48, 72) | 0 (0, 12) | 54 (39, 66) |
| Day 15 Post vaccination 2 | n | 61 | 61 | 27 | - |
|  | GMT (95% CI) | 345.4 (247.5, 482.1) | 2996.5 (2096.1, 4283.7) | 7.4 (-) | 8.7 (5.3, 14.1) |
|  | GMFR (95% CI) | 46.0 (32.8, 64.6) | 407.7 (285.2, 582.8) | 1.0 (-) | 8.9 (5.4, 14.4) |
|  | Seropositive % (95% CI), >= 10 titer | 100 (94, 100) | 98 (91, >99) | 0 (0, 13) | -2 (-9, 4) |
|  | Seroconversion % (95% CI), >= 4-fold rise | 97 (89, >99) | 98 (91, >99) | 0 (0, 13) | 2 (-6, 10) |
| Day 29 Post vaccination 2 | n | 62 | 62 | 28 | - |
|  | GMT (95% CI) | 243.3 (179.2, 330.3) | 2130.3 (1545.7, 2936.0) | 7.4 (-) | 8.8 (5.6, 13.6) |
|  | GMFR (95% CI) | 32.4 (23.7, 44.3) | 289.8 (210.3, 399.4) | 1.0 (-) | 8.9 (5.7, 13.9) |
|  | Seropositive % (95% CI), >= 10 titer | 100 (94, 100) | 100 (94, 100) | 0 (0, 12) | - |
|  | Seroconversion % (95% CI), >= 4-fold rise | 97 (89, >99) | 100 (94, 100) | 0 (0, 12) | 3 (-3, 11) |
| Day 85 Post vaccination 2 | n | 58 | 60 | 27 | - |
|  | GMT (95% CI) | 92.7 (65.8, 130.5) | 925.1 (645.0, 1326.9) | 7.4 (-) | 10.0 (6.1, 16.3) |
|  | GMFR (95% CI) | 12.3 (8.7, 17.4) | 125.9 (87.7, 180.5) | 1.0 (-) | 10.2 (6.2, 16.8) |
|  | Seropositive % (95% CI), >= 10 titer | 95 (86, 99) | 100 (94, 100) | 0 (0, 13) | 5 (-1, 14) |
|  | Seroconversion % (95% CI), >= 4-fold rise | 79 (67, 89) | 100 (94, 100) | 0 (0, 13) | 21 (11, 33) |
| Day 169 Post vaccination 2 | n | 59 | 57 | 28 | - |
|  | GMT (95% CI) | 51.8 (36.7, 73.1) | 466.6 (313.9, 693.5) | 7.4 (-) | 9.0 (5.4, 15.1) |
|  | GMFR (95% CI) | 6.9 (4.9, 9.8) | 63.5 (42.7, 94.4) | 1.0 (-) | 9.2 (5.5, 15.5) |
|  | Seropositive % (95% CI), >= 10 titer | 85 (73, 93) | 100 (94, 100) | 0 (0, 12) | 15 (7, 27) |
|  | Seroconversion % (95% CI), >= 4-fold rise | 64 (51, 76) | 95 (85, 99) | 0 (0, 12) | 30 (15, 45) |
| Peak Titer | n | 72 | 71 | 30 | - |
|  | GMT (95% CI) | 226.8 (154.1, 333.6) | 1865.2 (1215.9, 2861.3) | 7.4 (-) | 8.2 (4.6, 14.6) |
|  | GMFR (95% CI) | 30.3 (20.5, 44.7) | 253.8 (165.4, 389.3) | 1.0 (-) | 8.4 (4.7, 14.8) |
|  | Seropositive % (95% CI), >= 10 titer | 92 (83, 97) | 100 (95, 100) | 0 (0, 12) | 8 (2, 17) |
|  | Seroconversion % (95% CI), >= 4-fold rise | 89 (79, 95) | 99 (92, >99) | 0 (0, 12) | 10 (2, 19) |

Notes: N=Number of subjects in the Immunogenicity Population. n=Number of subjects with non-missing endpoint data.

*Day of Second Dose MV-CHIK vaccine, Sample taken pre-dose. For Cohorts 1 and 4, Day 29 Post vaccination 1 and Pre-vaccination Dose 2 are the same study time point.

†Difference = difference in the GMT, GMFR, Seropositive and Seroconversion rates, with 95% CI of the difference between Low Dose and High Dose MV-CHIK.

Exact 95% CIs for proportions are calculated using the Clopper-Pearson method. For the difference in proportions, exact unconditional confidence limits are calculated based on score statistics. 95% CIs are based on the t-test for differences in GMT and GMFR results.
