# Supplementary material for: A Phase 1 Double-Blinded Trial to Evaluate Safety, Immunogenicity, and Dosing of Measles-Vectored Chikungunya Virus Vaccine (MV-CHIK) in Healthy Adults
Source: J Infect Dis. 2025 Nov 28;233(3):e641–5. doi: 10.1093/infdis/jiaf571 (PMC13017142; doi:10.1093/infdis/jiaf571)
Supplement: jiaf571_Supplementary_Data [file jiaf571_supplementary_data.zip › Supplementary Table 4.docx]

Supplementary Table 4: Anti-CHIKV PRNT50 Antibody Results on Day 29 After Second Vaccination by Dose and Schedule (Immunogenicity Population)

|  | **Day 1- 29 (N=48)** | **Day 1- 85 (N=48)** | **Day 1- 169 (N=48)** | **P-Value^a^** |
| --- | --- | --- | --- | --- |
| **Low Dose MV-CHIK** | | | | |
| n | 21 | 22 | 19 | - |
| GMT (95% CI) | 80.0 (46.3, 138.2) | 99.7 (63.7, 156.2) | 107.1 (57.4, 199.7) | 0.709 |
| GMFR (95% CI) | 16.0 (9.3, 27.6) | 19.9 (12.7, 31.2) | 21.4 (11.5, 39.9) | 0.709 |
| Seropositive % (95% CI),  >= 10 titer | 100 (84, 100) | 100 (85, 100) | 100 (82, 100) | - |
| Seroconversion % (95% CI), >= 4-fold rise | 95 (76, >99) | 100 (85, 100) | 95 (74, >99) | 0.535 |
| **High Dose MV-CHIK** | | | | |
| n | 22 | 24 | 16 | - |
| GMT (95% CI) | 241.0 (145.0, 400.4) | 678.1 (426.3, 1078.5) | 794.8 (316.2, 1998.0) | 0.009 |
| GMFR (95% CI) | 48.2 (29.0, 80.1) | 135.6 (85.3, 215.7) | 159.0 (63.2, 399.6) | 0.009 |
| Seropositive % (95% CI),  >= 10 titer | 100 (85, 100) | 100 (86, 100) | 94 (70, >99) | 0.258 |
| Seroconversion % (95% CI), >= 4-fold rise | 100 (85, 100) | 100 (86, 100) | 94 (70, >99) | 0.258 |

N=Number of subjects in the Immunogenicity Population; n=number of subjects with non-missing data. Exact 95% CIs for proportions are calculated using the Clopper-Pearson method.

aP values of the differences between schedules for seropositive and seroconversion rates are calculated with Fisher's Exact test. P values of the difference between schedules for GMT and GMFR are based on ANOVA.
