# Supplementary figures and images for: A Phase 1 Double-Blinded Trial to Evaluate Safety, Immunogenicity, and Dosing of Measles-Vectored Chikungunya Virus Vaccine (MV-CHIK) in Healthy Adults
Source: J Infect Dis. 2025 Nov 28;233(3):e641–5. doi: 10.1093/infdis/jiaf571 (PMC13017142; doi:10.1093/infdis/jiaf571)

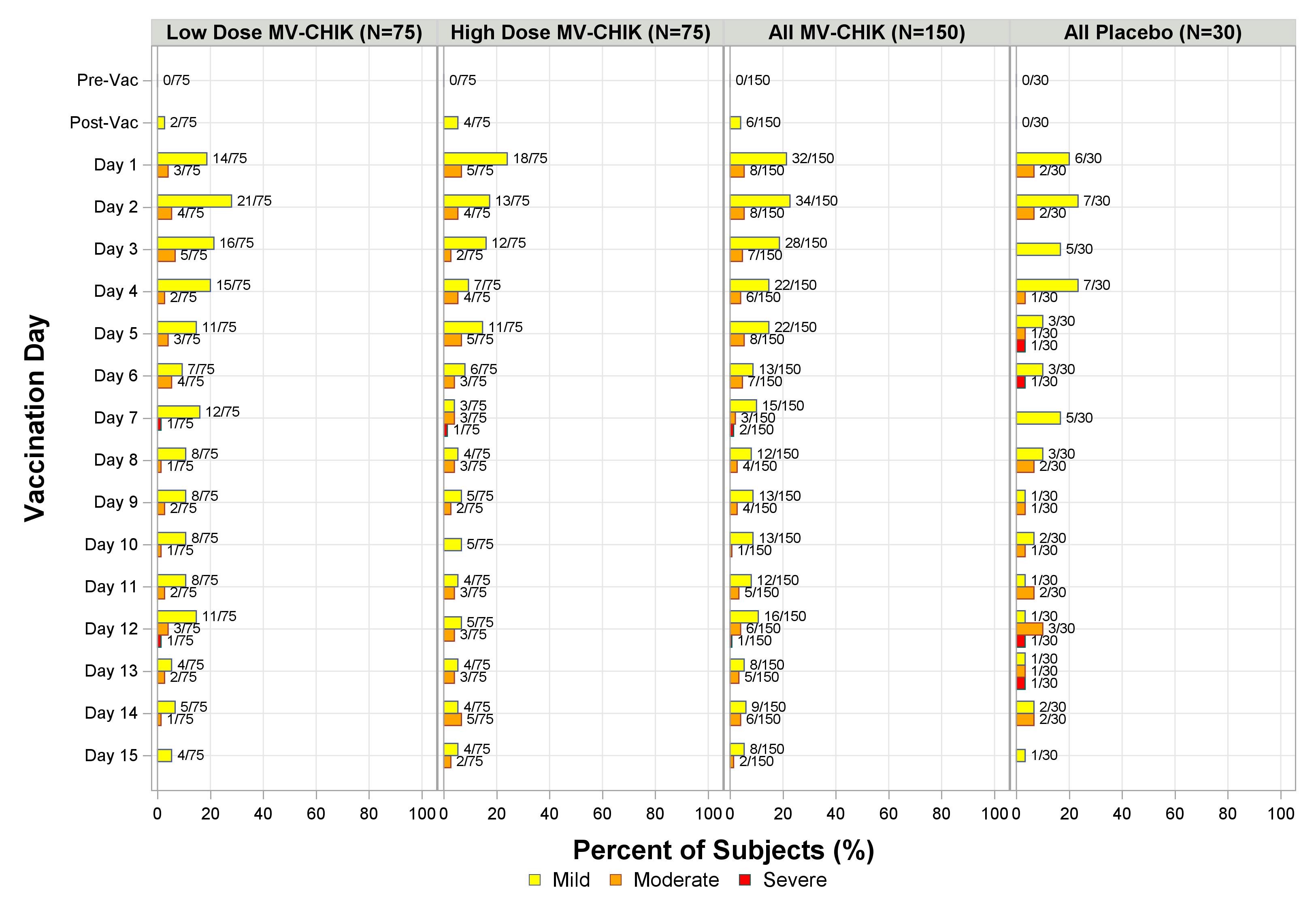

Supplement: jiaf571_Supplementary_Data [file jiaf571_supplementary_data.zip › SuppFig2_max sev systemic AE by day_MVCHIK.jpg]

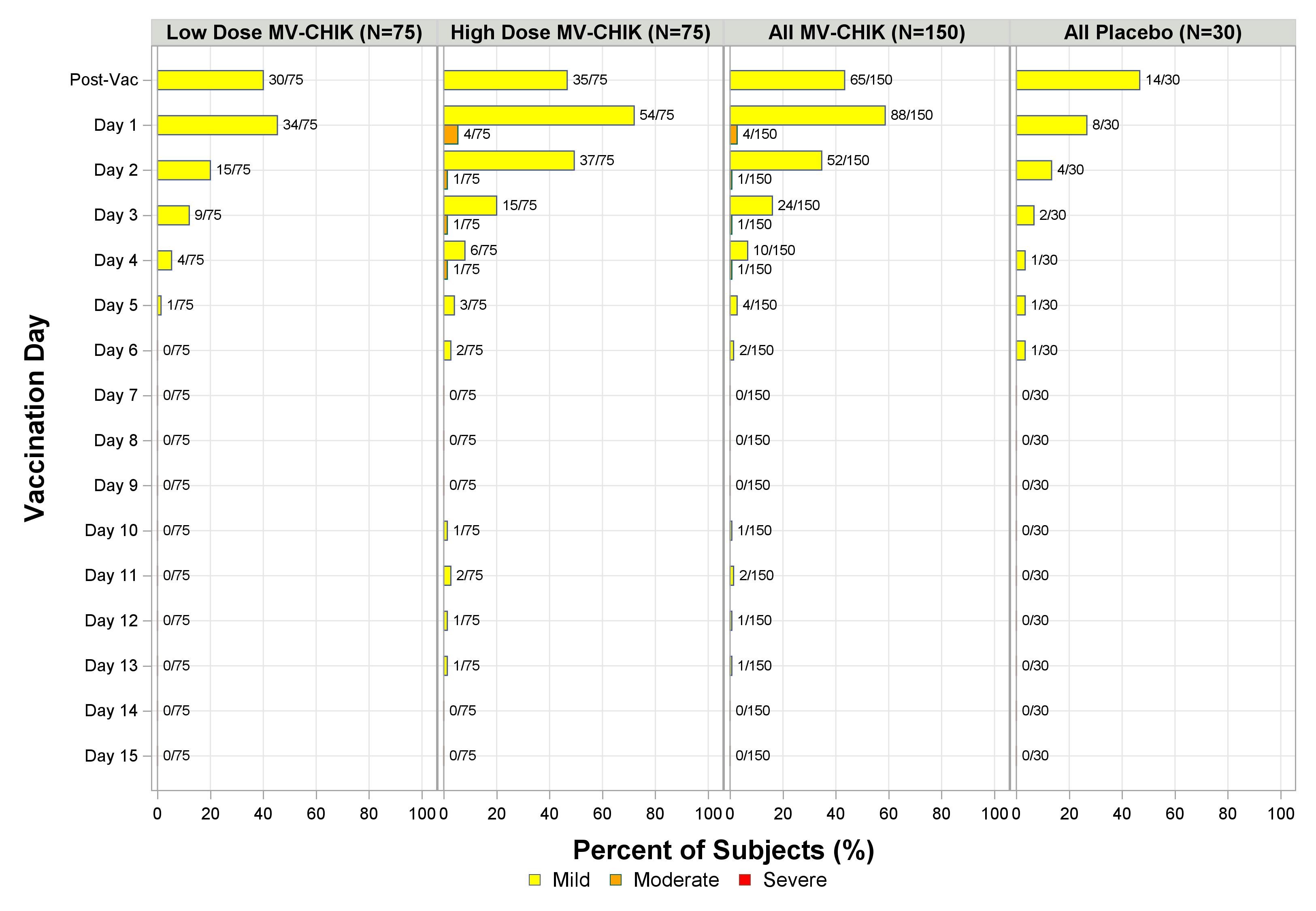

Supplement: jiaf571_Supplementary_Data [file jiaf571_supplementary_data.zip › SuppFig3_max sev local AE by day_MVCHIK.jpg]
